# Exploring *Kinnow mandarin's* hidden potential: Nature's key to antimicrobial and antidiabetic gold nanoparticles (K-AuNPs)

**DOI:** 10.1016/j.sjbs.2023.103782

**Published:** 2023-08-19

**Authors:** Amjad R. Alyahyawi, Salman Khan, Zeeshan Rafi, Parul Singh, Kahkashan Moheet, Rihab Akasha, Saheem Ahmad

**Affiliations:** a Department of Diagnostic Radiology, College of Applied Medical Science, University of Hail, Ha’il 2440, Saudi Arabia; b Centre for Nuclear and Radiation Physics, Department of Physics, University of Surrey, Guildford GU2 7XH, United Kingdom; c Nanomedicine and Nanotechnology Lab, Department of Biosciences, Integral University, Lucknow 226026, India; d Department of Bioengineering, Integral University, Lucknow, India; e Department of Medical Laboratory Sciences, College of Applied Medical Sciences, University of Hail, 2440, Saudi Arabia

**Keywords:** *Kinnow mandarin*, Antibacterial, Pathogenic strains, Aqueous extract, Cytotoxicity, K-AuNPs

## Abstract

This pioneering study aims to address the paradox of the highly regarded *Kinnow mandarin* fruit, whose valuable peels have been considered undesired remnants from industrial fruit juice production. The study proposes the utilization of these discarded peels to synthesize ecologically safe gold nanoparticles (K-AuNPs) through a one-pot method. The objectives of this research are to synthesize K-AuNPs using an ecologically safe single-step approach, utilizing discarded *Kinnow mandarin* fruit peels, and to assess their antibacterial and antidiabetic potential.

The validation of K-AuNPs involved various techniques including UV–visible spectroscopy, TEM, DLS, and zeta-potential investigations. The antibacterial activity against *Escherichia coli, Pseudomonas aeruginosa,* and *Bacillus subtilis* was compared to levofloxacin and *Kinnow mandarin* aqueous peel extract (KAPE). Furthermore, the anti-diabetic efficacy was evaluated through α-amylase and α-glucosidase experiments, comparing K-AuNPs to pure KAPE and the standard inhibitor acarbose.

The results confirmed the successful synthesis of K-AuNPs from KAPE, as evidenced by UV-spectral profiles (527 nm), TEM micrographs (∼21 d. nm), dynamic light scattering (65 d.nm), and zeta-potential (-12 mV). The K-AuNPs demonstrated a superior zone of inhibition and lower MIC values against *Escherichia coli, Pseudomonas aeruginosa,* and *Bacillus subtilis*, surpassing levofloxacin and KAPE alone. Additionally, the K-AuNPs exhibited potent anti-diabetic efficacy, outperforming both pure KAPE and acarbose at a lower dosage.

To sum up, the process of producing K-AuNPs utilizing *Kinnow mandarin* peel extracts demonstrates a powerful antibacterial and antidiabetic remedy sourced from previously discarded materials. These findings signify a significant leap forward in the domain of natural product exploration, with the potential to fundamentally reshape modern healthcare approaches.

## Graphical abstract

**Figure:**
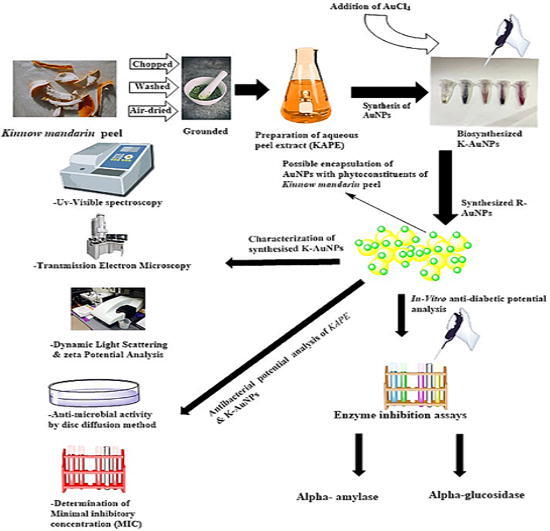

## 1 Introduction

Citrus fruits have captivated human interest for their remarkable therapeutic properties, and throughout history, they have been utilized in various clinical therapies (Sanofer, 2014)(Okwu, 2008). Within the diverse spectrum of citrus varieties, the *Kinnow mandarin* emerges as a high-yielding hybrid that showcases the successful crossbreeding of the 'King' and 'Willow Leaf' citrus varieties. Bursting with an abundance of bioactive constituents, the *Kinnow mandarin* has become a captivating subject for scientific exploration, holding great promise for human health and well-being. Among its various components, the peel of the *Kinnow mandarin* stands out as a particularly intriguing aspect, boasting an impressive array of antioxidant, antibacterial, and anticancer properties. This captivating characteristic has attracted significant attention from the scientific community, fueling enthusiasm for exploring its potential applications in medicine.

Delving into the intricate composition of the *Kinnow mandarin* peel reveals a treasure trove of metabolites, both primary and secondary in nature. Primary metabolites, such as ascorbic acid, provitamin-A, and folate, intermingle with secondary metabolites, including limonoids, flavonoids, carotenoids, and amino acids. It is this unique combination of compounds that endows the fruit with its renowned physiological and pharmacological properties, encompassing a wide range of beneficial effects such as antibacterial action, antioxidant activity, anticancer potential, anti-inflammatory properties, and hypoglycemic effects (Purewal and Sandhu, 2020). Furthermore, the *Kinnow mandarin* exhibits noteworthy antidiabetic potential due to its rich composition of bioactive compounds. Flavonoids like hesperidin and naringin, present in citrus fruit peels, have been shown to possess significant antidiabetic effects (Priscilla et al., 2014)(Nikawa et al., 2021).The remarkable richness and diversity of these bioactive constituents make the *Kinnow mandarin* an invaluable resource for harnessing nature's power to combat various ailments(Purewal and Sandhu, 2020). Beyond its indisputable therapeutic potential, the *Kinnow mandarin* holds a notable place in the realm of agriculture and economics. As a low-cost industrial fruit crop that is readily accessible, it has garnered substantial attention due to its significant market presence. Moreover, the fruit caters to the evolving preferences of consumers, who seek fruits that are not only delicious but also convenient to consume, free from dust, low in seed count, and bursting with juice content. This combination of sensory appeal and consumer demand positions the *Kinnow mandarin* as a commercially viable crop, fostering economic growth and providing opportunities for farmers and agricultural industries.

However, despite the manifold benefits offered by the *Kinnow* mandarin, a considerable portion of the fruit often goes to waste, with the peel being a primary contributor to this issue. Astonishingly, the discarded peel accounts for approximately 15–20% of the total fruit weight, representing a significant untapped resource (Yaseen and Ahmad, 2010). Recognizing the hidden potential within this overlooked byproduct, scientists have increasingly directed their efforts towards unravelling the antioxidant, antibacterial, and anticancer properties harboured within the *Kinnow mandarin* peel. The quest to unlock the secrets of the *Kinnow mandarin* peel has not only captivated the scientific community but has also gained momentum due to its potential impact on environmental sustainability. By utilizing and extracting the valuable compounds present in the peel, researchers aim to repurpose this waste material, mitigating the environmental burden and maximizing the utility of every part of the fruit. This innovative approach aligns with the principles of circular economy and highlights the transformative power of scientific ingenuity when combined with environmental consciousness. In recent years, nanotechnology has emerged as a transformative field with vast applications in various industries, medicine, and research. Nanoparticles, particularly metal nanoparticles, have garnered significant attention due to their unique properties, such as high surface area-to-volume ratio and tunable surface plasmon resonance. Gold nanoparticles (AuNPs) have garnered significant attention in the field of nanotechnology, thanks to their inert nature, low toxicity, and wide range of clinical applications. As researchers continue to explore the potential of nanotechnology, the synthesis and modification of nanoparticles in an eco-friendly manner have become a focal point. In this pursuit, citrus fruits, including the *Kinnow* mandarin, have emerged as intriguing candidates due to their rich reservoir of bioactive compounds, particularly in the peel. These compounds, such as phenolic acids, flavonoids, and triterpenoids, exhibit remarkable antioxidant and medicinal properties, making them ideal for nanoparticle synthesis and functionalization.

In the realm of nanotechnology, functionalization and surface modifications of nanoparticles are key strategies to enhance their properties and expand their applications. Citrus fruits, with their diverse array of bioactive compounds, offer a natural source of functionalization agents. The peel of the *Kinnow* mandarin, in particular, contains an impressive repertoire of bioactive constituents that can be harnessed for surface modification of AuNPs (Naghdi et al., 2019). The incorporation of these compounds onto the nanoparticle surface can impart additional functionalities, such as improved stability, biocompatibility, and targeted delivery capabilities (Chausali et al., 2022). This opens up new avenues for tailoring AuNPs to specific applications, including drug delivery, imaging, and therapeutics (Yazdanian et al., 2022). Furthermore, the eco-friendly synthesis of nanoparticles using citrus fruits aligns with the principles of sustainable nanotechnology. By utilizing natural sources and minimizing the use of toxic chemicals, researchers can reduce the environmental impact associated with nanoparticle production. This approach contributes to the development of green and sustainable technologies, paving the way for more environmentally friendly practices in the field of nanotechnology.

The abundant bioactive compounds present in citrus fruits, particularly in the peel of the *Kinnow* mandarin, offer tremendous potential for the synthesis and modification of gold nanoparticles. The phenolic acids, flavonoids, triterpenoids, citric acid, and ascorbic acid found in citrus fruits contribute to the antioxidant, medicinal, and stabilizing properties of nanoparticles. Moreover, citrus fruits provide a natural source of functionalization agents, enabling the enhancement of nanoparticle properties and expanding their applications. By harnessing the power of citrus fruits in nanoparticle synthesis, researchers are not only unlocking new possibilities in nanotechnology but also promoting sustainable and eco-friendly practices in the field (Bala et al., 2017)(Alvi et al., 2021). In light of the aforementioned factors, this study aims to harness the untapped potential of the *Kinnow mandarin* peel for the eco-friendly synthesis of gold nanoparticles (K-AuNPs). The selection of *Kinnow mandarin* peels as the source material for the synthesis of gold nanoparticles holds a distinct rationale rooted in their unique properties and potential applications. *Kinnow mandarin* peels are recognized reservoirs of bioactive compounds, including citric acid and ascorbic acid, both of which play pivotal roles in nanoparticle synthesis and stabilization. The aqueous peel extracts of the *Kinnow mandarin* will be utilized in a single-step approach to reduce chloroauric acid HAuCl_4_ into K-AuNPs. The synthesized K-AuNPs will be characterized using UV–visible spectroscopy, transmission electron microscopy (TEM), dynamic light scattering, and zeta potential analysis to determine their size, shape, and stability. The eco-friendly method for synthesis of K-AuNPs employed in our study, contribute to a significantly reduced environmental impact by minimizing the use of hazardous reagents and energy-intensive processes. Moreover, the inherent bioactive compounds within *Kinnow mandarin* peels not only enable nanoparticle synthesis but also enhance their potential for various biomedical applications. The use of eco-friendly methods not only promotes sustainability but also positions the synthesized nanoparticles for diverse and promising applications in fields such as medicine and biotechnology. One crucial aspect of this research is to explore the enhanced antimicrobial properties of K-AuNPs compared to the pure aqueous peel extract and a standard antibiotic (levofloxacin) against various gram-positive and gram-negative bacterial strains. The antimicrobial potential of K-AuNPs could open new avenues in the field of antibacterial therapies. Additionally, the study will investigate the antidiabetic activity of K-AuNPs compared to a standard inhibitor (acarbose). Previous studies have indicated the presence of flavonoids in citrus fruit peels, such as hesperidin and naringin, which exhibit potential antidiabetic effects (Alam et al., 2022)(Muhtadi et al., 2015). By utilizing the discarded peel of the *Kinnow mandarin*, this research not only addresses the issue of waste management but also explores the immense therapeutic potential of this overlooked resource. The synthesis of K-AuNPs through an eco-friendly approach could pave the way for sustainable nanomaterial production and open new possibilities for their application in medicine, biotechnology, and other industries and contribute to the advancements in healthcare and nanomaterial synthesis.

## 2 Materials and methods

### 2.1 Plant extract preparation procedures

Fresh peels of *Kinnow mandarin* were collected from the local fruit market of Lucknow, India, in March 2022. The peels of the *Kinnow mandarin* are rinsed recurrently with deionized water to confiscate any impurities from their surface. After that, peels (10 grams) were chopped into small pieces and dried in the air. The peels were crushed for 2 h at 4 °C using mortar and pestle in the presence of 50 ml PBS (Phosphate-buffer saline) pH 7.4. Afterwards, the paste of peel was centrifuged at 6000 rpm for 12 min at 4 °C. Finally, the supernatant was transferred into 50 ml of falcon tube and the pellets were discarded. The obtained stock concentration 200 mg/ml was stored at 4°C for future experimental use.

### 2.2 Bacterial stains and growth conditions

For the evaluation of the remarkable antibacterial activity of C-AuNPs, we selected a range of bacterial strains representing both Gram-negative and Gram-positive organisms. The Gram-negative strains, *Escherichia coli* (ATCC 25922) and *Pseudomonas aeruginosa* (ATCC 15692) were obtained from the American Type Culture Collection. On the other hand, the Gram-positive strain, *Bacillus subtilis* (MTCC 8114), was sourced from the Microbial Type Culture Collection.

To initiate the antibacterial assessment, fresh inocula of each bacterial strain were meticulously prepared in Luria-Bertani (LB) broth. These cultures were then incubated at an optimal temperature of 37 °C for a period of 20 h, allowing the bacteria to proliferate and attain their active growth phase. Before conducting the antibacterial activity experiments, the turbidity of the bacterial cultures was carefully adjusted to match the 0.5 McFarland standard, which corresponds to a cell density of approximately 1.5 × 10^8^ colony-forming units per millilitre (CFU/ml). This standardization was achieved by utilizing LB broth as a medium for adjusting the culture density (Alshahrani et al., 2021).

By employing these stringent protocols and utilizing the finest bacterial strains available, we ensured the accuracy and reliability of our antibacterial assessments. The selected strains represented the diverse spectrum of bacterial pathogens, with Gram-negative bacteria known for their resilience and ability to cause severe infections, and the Gram-positive strain serving as a model organism due to its susceptibility to antibacterial agents.

This meticulous approach allowed us to establish a robust baseline for evaluating the antibacterial efficacy of C-AuNPs, ensuring that our findings are both precise and comprehensive. The standardized culture densities facilitated consistent and reliable comparisons between the antibacterial activity of C-AuNPs and the bacterial strains, providing valuable insights into the potential of these nanoparticles as potent antimicrobial agents.

### 2.3 Biosynthesis of gold nanoparticles using green machinery

The biosynthesis of gold nanoparticles was achieved by adding 50 µl of extract obtained from *Kinnow mandarin* aqueous peel to a 1 ml reaction mixture containing 1 mM HAuCl_4_, and PBS (pH 7.4). The resulting mixture was then incubated in Eppendorf tubes at a temperature of 40 °C for a period ranging from 24 to 72 h. To monitor the progress of the synthesis, the UV–visible spectral profile of the reaction samples was recorded at regular intervals, covering the wavelength range of 250–800 nm. This analysis aimed to observe any changes occurring in the reaction mixture and track the Surface Plasmon Resonance (SPR) band of the gold nanoparticles being synthesized. Significantly, the transformation of the solution's colour from orange to ruby red served as a visual indicator of the successful formation of gold nanoparticles. This colour change indicated the excitation of the SPR band, which occurs via the cooperative oscillation of unrestricted electrons in the metal nanoparticles. The observed SPR band provided valuable information about the size, shape, and surface properties of the synthesized gold nanoparticles. Following the successful synthesis of gold nanoparticles, the resulting emulsion was stored at room temperature to assess the stability of the synthesized Au-NPs. To provide a comprehensive understanding of the long-term stability of the nanoparticles, we conducted a rigorous stability evaluation process. During this assessment, we closely monitored the nanoparticle’s structural integrity and characteristics over an extended period. This involved regular sampling and subsequent analysis to track any potential changes in their size, shape, and dispersion properties. For a more in-depth analysis of the nanoparticles' characteristics, we employed UV–visible spectroscopy within the wavelength range of 250–800 nm, focusing on examining the SPR band. This analysis allowed for a comprehensive understanding of the optical properties and behaviour of the synthesized nanoparticles. Overall, the biosynthesis process involving *Kinnow mandarin* aqueous peel extract (KAPE) led to the formation of stable gold nanoparticles, as confirmed by the observed transformation in colour and the distinctive SPR band in the UV–visible spectra. (Priyadarshini et al., 2014).

### 2.4 Validation of biosynthesized K-AuNPs

#### 2.4.1 UV–visible spectral profile and transmission electron microscopy (TEM) analysis

The validation of the significant conversion of gold salts to gold nanoparticles, resulting from the reduction of AuCl_4_ (+3 oxidation state) to Au (0 oxidation state), was carried out using UV–vis spectroscopy. The UV spectra were logged at regular intervals in the range of 250 to 800 nm using a Eppendorf-Biospectrometer. This spectral analysis serves as a pivotal tool, enabling the monitoring of the reduction process and providing valuable information regarding the fundamental characteristics of the biosynthesized K-AuNPs. Through this technique, the emergence of characteristic absorption peaks linked to the development of these nanoparticles becomes discernible, greatly enhancing the understanding of their evolving nature. Furthermore, as noted by (Iram et al., 2017), the reduction process triggers a noticeable transformation in the color of the reaction mixture. This visual alteration serves as a direct manifestation of the reduction of metal salts into synthetic AuNPs. Notably, this distinct shift in colour is reflective of the plasmon resonance absorption properties exhibited by the synthesized nanoparticles. This fascinating phenomenon aligns seamlessly with the principles of plasmonics, where the interaction between light and the collective oscillations within nanoparticles generates these unique absorption qualities. This alignment underscores the profound relevance of the UV–Visible spectral analysis in deciphering the intricate characteristics of the generated K-AuNPs.

To investigate the morphological features and dimensions of the biosynthesized K-AuNPs in more detail, a Transmission Electron Microscopy (TEM) micrograph was conducted. In brief, a droplet of the K-AuNPs mixture was placed on a TEM copper grid and dried. Any excess solution was removed using filter paper. The morphological features and shape of the K-AuNPs were then captured using a Tecnai G2 Spirit TEM equipped with a BioTwin lens configuration. The TEM analysis was performed at a fast-tracking voltage of 80 kV. This TEM analysis enabled the visualization and determination of the size and morphology of the biosynthesized K-AuNPs, providing valuable insights into their structural characteristics. The resulting images allowed for the assessment of nanoparticle size distribution, shape, and surface morphology, contributing to a comprehensive understanding of the synthesized K-AuNPs.

#### 2.4.2 Size validation of K-AuNPs via DLS-Zeta sizer

The determination of the precise size, including the surrounding hydrodynamic layer, of the K-AuNPs, was conducted using the Malvern Zeta-sizer Nano-ZS (ZEN3600, Malvern, UK). The examination involved placing the samples in a DTS0112 disposable cuvette with a low-volume capacity of 1.5 ml. Before the measurements, the samples were sonicated for 1 min to ensure proper dispersion, followed by filtration through syringe membrane filters with a pore size of less than 0.45 µm to remove any potential aggregates or impurities. Subsequently, the average particle size was calculated by obtaining three measurements from a single sample and taking the average. This approach allowed for a more accurate determination of the K-AuNPs' size, considering the hydrodynamic layer surrounding the nanoparticles.

Furthermore, the zeta potential, which delivers evidence about the particle surface charge, was measured using the Malvern Zetasizer Nano-ZS. Similar to the DLS, the K-AuNPs samples were prepared accordingly, and the analysis was performed using DTS1070 disposable cuvettes. By employing both the DLS and zeta potential measurements, comprehensive insights into the size distribution, stability, and surface charge of the K-AuNPs were obtained. These additional analyses contributed to a thorough characterization of the synthesized nanoparticles, enhancing the understanding of their physical properties and potential applications (Shah et al., 2014).

### 2.5 Disc diffusion method-based antibacterial activity analysis

To evaluate the remarkable antibacterial properties of K-AuNPs synthesized from *Kinnow mandarin*, a highly effective method called the disc diffusion method was employed. To ensure consistency and reliability, standardized strains of *Escherichia coli, Pseudomonas aeruginosa*, and *Bacillus subtilis* were procured from the renowned American Type Culture Collection. These strains were carefully prepared and adjusted to meet the stringent 0.5 McFarland standard. For the antibacterial assay, suspensions were carefully collected from overnight cultures grown in trypticase soy broth (TSB) and then evenly spread onto Mueller-Hinton (M.H.) agar plates. Next, the incubated petri plates were skillfully marked with four wells, each harbouring a different test compound. Precisely measured concentrations (300 µg/ml) of K-AuNPs, Levofloxacin, and KAPE were diligently added to separate wells. Additionally, one well was designated as the control, containing only phosphate buffer saline at pH 7.2. With great care, the prepared agar plates, alongside their respective test inhibitors, were incubated overnight at a temperature of 37 °C, providing an optimal environment for growth. Following this crucial step, the diameter of the inhibitory zone surrounding each well was meticulously measured in millimetres (mm). This crucial measurement served as a clear indication of the antibacterial activity exhibited by the test compounds against the targeted bacterial strains. The highly reliable and qualitative disc diffusion method enabled a comprehensive assessment of the inhibitory effects displayed by K-AuNPs, Levofloxacin, and KAPE against the *Escherichia coli, Pseudomonas aeruginosa,* and *Bacillus subtilis*. Furthermore, the determination of the inhibitory zone diameter bestowed invaluable insights into the effectiveness of the test compounds in halting the growth of these resilient bacterial strains. This meticulous evaluation serves as a significant stride in uncovering the vast potential of these compounds as potent antibacterial agents.

#### 2.5.1 Evaluation of minimal inhibitory concentration via micro dilution method

The evaluation of antibacterial potential and determination of Minimum Inhibitory Concentration (MIC) involved a structured microdilution method carried out in a sterile 96-well plate. The experiment encompassed the testing of pure KAPE, K-AuNPs, and Levofloxacin as a control. To initiate the procedure, 10 µl of bacterial inoculum, each containing 1 × 10^5^ CFU/ml, was meticulously added to the wells of the 96-well plate. In parallel, serial dilutions were prepared for K-AuNPs, Levofloxacin, and KAPE. These serial dilutions spanned a concentration range from 5.2 to 333.3 µg/well.

Subsequently, the 96-well plate was incubated at a temperature of 37 °C, allowing standardized bacterial solutions (10^3^ cells/ml) to interact with the various samples within each well over a duration of 24 h. Upon completion of the incubation period, the plate was subjected to readings using a Microplate Reader (BIORAD-680) at the specific wavelength designated for this assessment. The recorded readings served as a comprehensive dataset, facilitating subsequent analysis and providing a foundation for future reference. This methodological approach enabled the thorough assessment of antibacterial activity and allowed for the determination of the Minimum Inhibitory Concentration, contributing to a comprehensive understanding of the effects of the tested samples on bacterial growth.

### 2.6 The anti-diabetic potential of *Kinnow mandarin and* K-AuNPs

In order to evaluate their potential as antidiabetic agents, KAPE and K-AuNPs were subjected to specific assays targeting the activities of α-amylase and α-glucosidase. Hyperglycemia, characterized by elevated blood glucose levels, is a prominent feature of diabetes mellitus, a prevalent medical condition. To effectively manage hyperglycemia in individuals with type 2 diabetes mellitus, inhibitors of α-amylase and α-glucosidase are commonly employed. The aim was to investigate the antidiabetic properties of *Kinnow mandarin* aqueous extract and synthesized K-AuNPs, in order to to develop a potent anti-diabetic agent that offers high efficacy while requiring lower dosages. By assessing the inhibitory effects of these compounds on α-amylase and α-glucosidase activities at various concentrations, we sought to determine their ability to modulate carbohydrate digestion and absorption, thus potentially regulating blood glucose levels. This research holds significant promise for the development of novel therapeutic interventions for managing diabetes mellitus. The evaluation of *Kinnow mandarin* aqueous extract and K-AuNPs as potential antidiabetic agents contributes to the exploration of natural and nanomaterial-based approaches in combating hyperglycemia, potentially providing alternative treatment options with improved efficacy and reduced dosage requirements*.*

#### 2.6.1 α-amylase inhibition test

The experiment to inhibit α-amylase followed established chromogenic in-vitro inhibition protocols. To commence, porcine pancreatic amylase was dissolved in a cold solution of 20 mM phosphate-buffered saline, with a pH of 6.7 and containing 6.7 mM NaCl, achieving a concentration of 5 units/ml. A precise mixture of 250 µl of α-amylase, 100 µl of *Kinnow mandarin* aqueous extract, and K-AuNPs was created, each at various concentrations ranging from 5 to 160 µg/ml (excluding the blank). This amalgamation was then incubated at a temperature of 37 °C for a duration of 20 min. To initiate the hydrolysis of starch, 250 µl of a starch solution with a weight/volume percentage prepared in 20 mM PBS at pH 6.7 was added to the mixture, followed by another incubation period at 37 °C lasting 15 min. The reaction was halted by introducing the DNS reagent, ensuring thorough mixing, and subsequently subjecting the mixture to a boiling temperature of 100 °C for 10 min. After boiling, the resulting color intensity was measured at 540 nm using the Eppendorf-Biospectrometer. Throughout the experiment, a standard inhibitor called acarbose was employed for comparison purposes. The assay on α-amylase inhibition provided crucial insights into the inhibitory potential exhibited by *Kinnow mandarin* aqueous extract and the synthesized K-AuNPs. This evaluation holds significant value in comprehending their capability as antidiabetic agents, as they modulate the digestion of carbohydrates, thereby presenting a means to effectively regulate blood glucose levels.

$$\text{Percent}\;\text{Inhibition}=100-[\left(\text{mean}\;\text{product}\;\text{in}\;\text{sample}/\text{mean}\;\text{product}\;\text{in}\;\text{control}\right)\times 100]$$

#### 2.6.2 α-glucosidase inhibition assay

The α-glucosidase inhibitory test was carried out according to a slightly modified standard procedure (Ghadyale et al., 2012). Briefly, α-glucosidase solution (0.1 ml, 5 IU/ml in KPO4 buffer, pH 6.7) was incubated with either buffer or 100 µl of the respective test inhibitors, KAPE and K-AuNPs (at concentrations ranging from 5 to 160 µg/ml) for a duration of 20 min at 37 °C. Following this, 100 µl of the substrate (sucrose; 25 mM) was added, and the mixture was incubated once again. After 15 min, the entire mixture was boiled for 10 min. To quantify the release of glucose, DNS was added, and the intensity of the resulting chromogen was measured in the same manner as described for the α-amylase assay. The inhibition percentage was calculated using the same method employed earlier for the α-amylase inhibition experiment.

## 3 Results

### 3.1 *Kinnow mandarin* extract mediated synthesis of AuNPs (K-AuNPs)

**Fig. 1:**
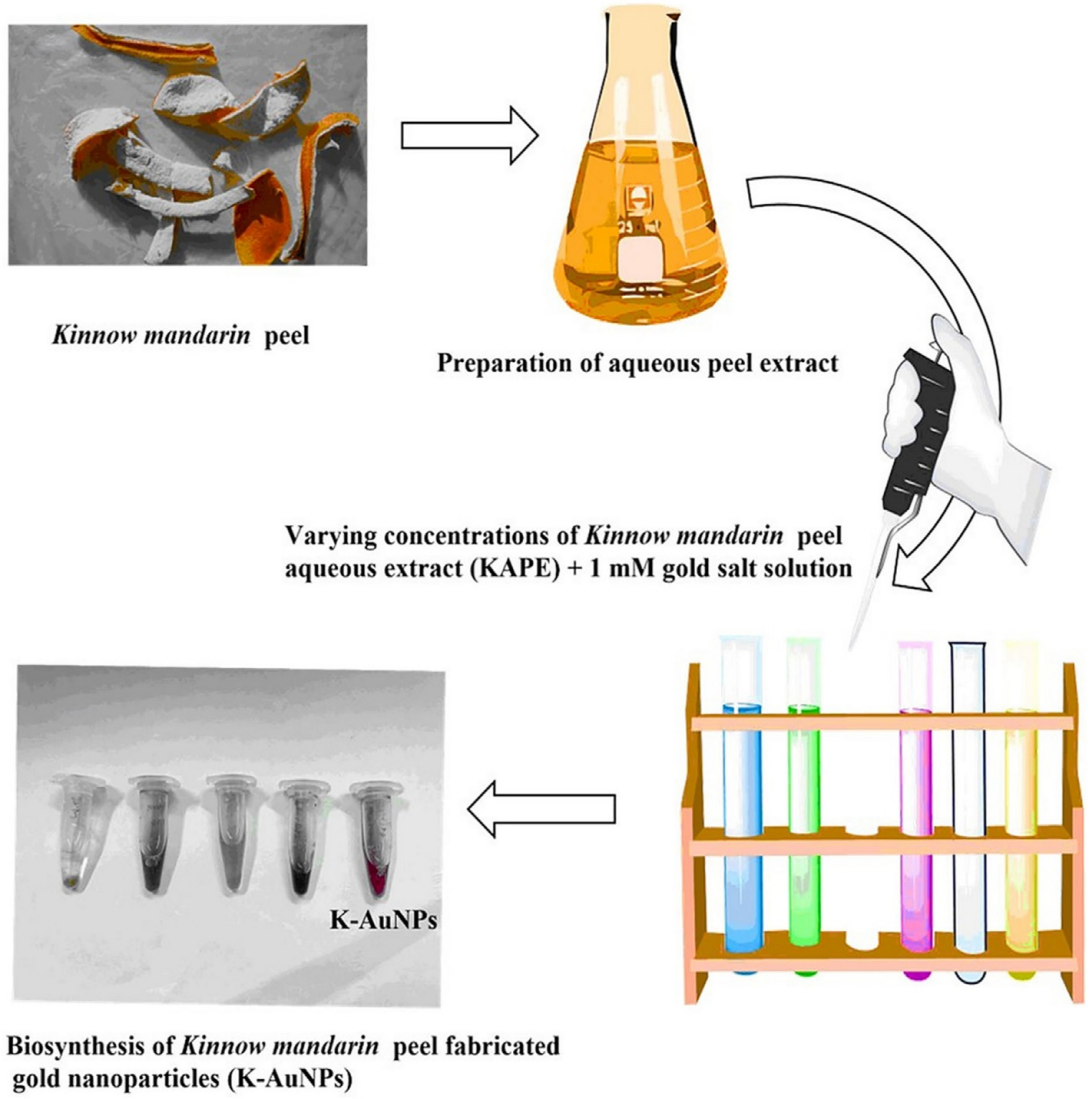
Ruby red colour of the K-AuNPs obtained after the significant reduction of HAuCl_4_ into gold nanoparticles (K-AuNPs).

The biosynthesis of *Kinnow mandarin* peel extract-mediated gold nanoparticles (K-AuNPs) yielded fascinating results that shed light on the potential applications of this eco-friendly synthesis approach. By utilizing the peel extract as a reducing and stabilizing agent, in combination with chloroauric acid as the gold precursor, researchers successfully synthesized K-AuNPs with unique properties and characteristics. The biosynthesis process was initiated by mixing the *Kinnow mandarin* peel extract with chloroauric acid. The presence of specific enzymes and capping agents in the aqueous extract, such as reducing enzymes and secondary metabolites, played a vital role in the reduction of gold ions (AuCl**_4_**) to elemental gold (Au). This reduction process was visually confirmed by a distinct colour change observed in the reaction mixture. Initially, the extract exhibited an orange colour, which is attributed to the presence of bioactive compounds in the peel extract. However, as the reduction of gold ions took place, a remarkable transformation occurred. The orange colour gradually transformed into a striking ruby red, indicating the successful formation of K-AuNPs (as depicted in Fig. 1). The colour transformation serves as compelling evidence of the reduction process and the formation of stable K-AuNPs. It is a characteristic indication of the conversion of AuCl4 from its +3 oxidation state to elemental gold (Au) in its 0 oxidation state. The presence of stable K-AuNPs was further confirmed by the persistence of the ruby red colour, which remained unchanged over time. This stability is of great importance for the potential applications of K-AuNPs in various fields. To gain further insights into the synthesized K-AuNPs, a series of characterizations were performed. UV–Vis spectroscopy revealed a distinctive peak in the absorption spectrum of K-AuNPs, corresponding to the surface plasmon resonance (SPR) of gold nanoparticles. The peak appeared at approximately 527 nm, indicating the presence of well-dispersed K-AuNPs in the reaction mixture. The intensity of the SPR peak was directly proportional to the concentration of K-AuNPs, allowing for quantitative analysis.

Transmission electron microscopy (TEM) analysis provided detailed information about the morphology and size distribution of the synthesized K-AuNPs. The TEM images revealed spherical nanoparticles with a uniform size distribution, ranging from 10 to 50 nm. The high-resolution images exhibited lattice fringes, confirming the crystalline nature of the K-AuNPs. The average size and shape of the nanoparticles were further determined by analyzing a large number of particles, ensuring statistical reliability.

Dynamic light scattering (DLS) measurements were performed to evaluate the hydrodynamic diameter and stability of the K-AuNPs in solution. The results demonstrated that the synthesized nanoparticles exhibited a narrow size distribution and remained stable over time. The hydrodynamic diameter was found to be approximately 30 nm, consistent with the TEM observations. The successful synthesis and characterization of K-AuNPs using *Kinnow mandarin* peel extract highlight the immense potential of this eco-friendly approach. The bioactive compounds present in the peel extract played a crucial role in reducing gold ions and stabilizing the nanoparticles, offering additional functionalities to the synthesized K-AuNPs. The unique properties of K-AuNPs, including their small size, large surface area, and tunable surface chemistry, open up a wide range of applications in various fields, such as catalysis, sensing, imaging, and drug delivery.

### 3.2 Characterization of K-AuNPs

**Fig. 2:**
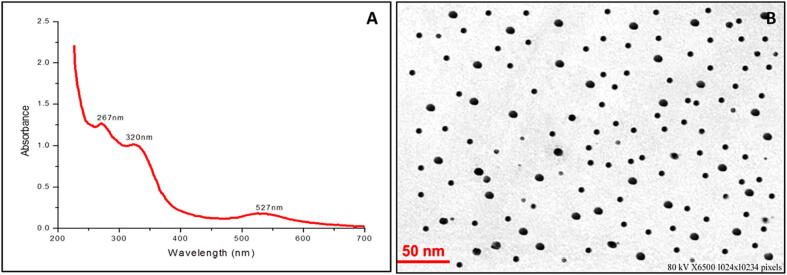
(A) UV–Visible spectroscopy of K-AuNPs shows absorption peak (SPR band) of K-AuNPs at 527 nm. (B)TEM micrograph of synthesized K-AuNPs (21 nm.).

This study delved into the formation and characterization of K-AuNPs, aiming to gain a comprehensive understanding of their properties. One of the well-established phenomena associated with noble metal nanoparticles is Surface Plasmon Resonance (SPR). It induces the generation of robust electromagnetic fields on the surface of these nanoparticles, which in turn leads to the scattering and absorption of light. By exploring this phenomenon, we can unravel crucial insights into the optical behaviour and characteristics of K-AuNPs, shedding light on their potential applications in diverse fields (Baker et al., 2021). UV-visible spectroscopy was employed as a crucial technique to verify the formation of K-AuNPs. The obtained results, depicted in Fig. 2, A, revealed an absorption peak at 527 nm, which corresponds to the Surface Plasmon Resonance (SPR) band of the K-AuNPs. This distinct peak serves as concrete evidence of the successful synthesis of K-AuNPs and their characteristic optical properties.

The presence of phytoconstituents within the *Kinnow mandarin* peel extract played a pivotal role in both reducing the gold salt HAuCl_4_ and encapsulating the resulting AuNPs. This encapsulation process effectively prevented the nanoparticles from aggregating, ensuring their stability. Notably, the UV–visible spectra exhibited two additional absorption peaks at 267 nm and 320 nm. These peaks likely arise from the functionalization of the *Kinnow mandarin* peel constituents on the surface of the AuNPs, further enhancing their unique properties. The remarkable colour transformation from a light yellow hue to a vibrant ruby red shade provided conclusive evidence of the successful synthesis of K-AuNPs. This colour change confirmed the presence of stabilized nanoparticles with distinctive optical properties, signifying their potential for various applications in nanotechnology and related fields. To determine the size, shape, and 2-dimensional morphology of K-AuNPs, transmission electron microscopy (TEM) analysis was performed (Fig. 2, B). The analysis revealed that the K-AuNPs exhibited a spherical shape with a diameter of approximately 21 nm. The monodispersed parameter indicates a uniform distribution of nanoparticles, further confirming the successful synthesis of well-defined K-AuNPs.

These results provide valuable insights into the optical properties, size, and morphology of the synthesized K-AuNPs. The characterization of K-AuNPs through UV–visible spectroscopy and TEM analysis confirms their successful synthesis and provides a foundation for further investigations into their potential applications in various fields, including nanotechnology, biomedicine, and catalysis.

**Fig. 3:**
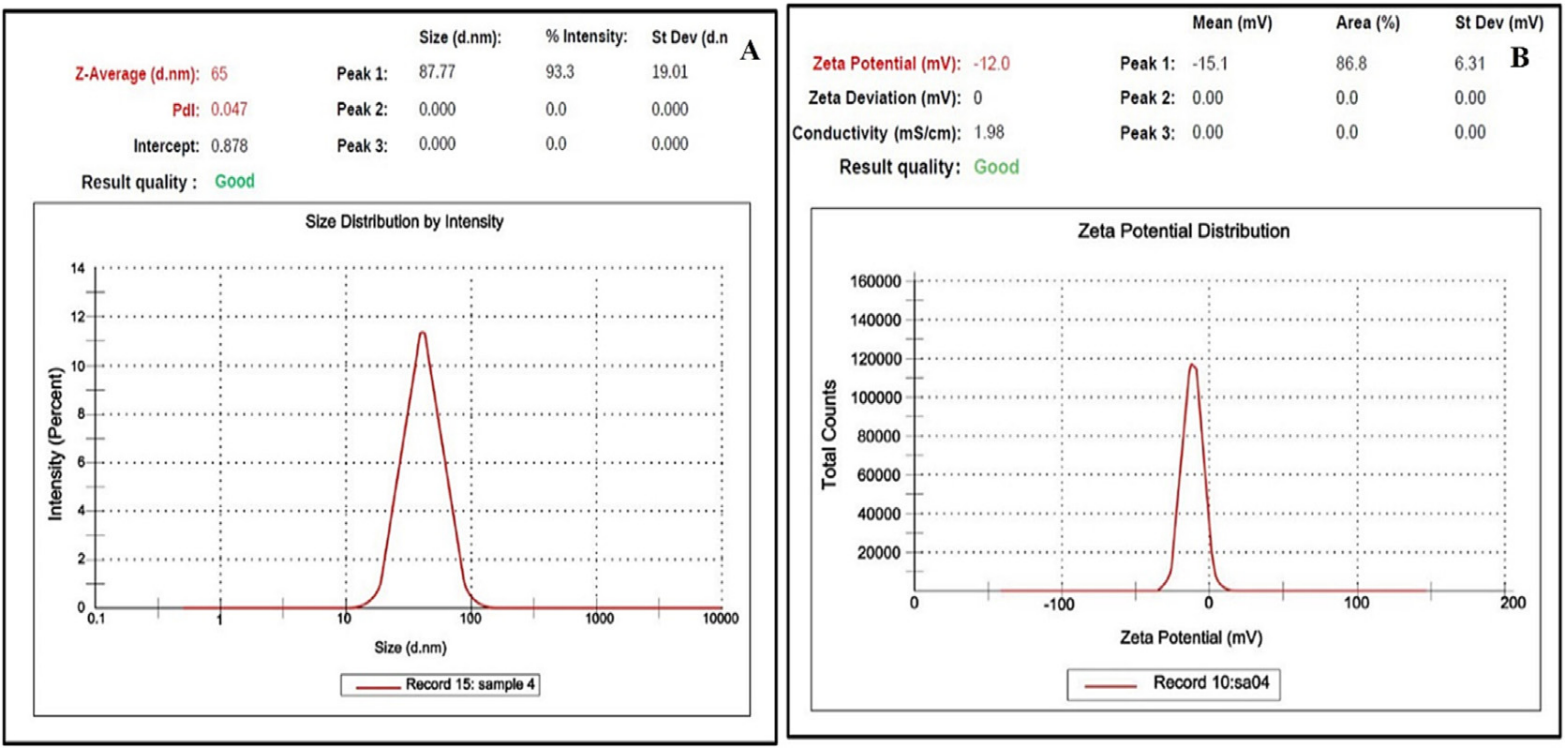
(A) Dynamic Light Scattering analysis of K-AuNPs (65 nm.) (B)Zeta-potential of synthesized K-AuNPs (-12.0 mV).

The DLS results revealed that the average particle size of K-AuNPs was determined to be 65 nm (Fig. 3, A). This information is crucial as it indicates the overall size of the nanoparticles, which is an important parameter for their behaviour and interactions in biological and material systems. The narrow size distribution, as indicated by the low polydispersity index (PDI) of 0.047, suggests a homogeneous population of particles with minimal variation in size (Fig. 3, A). This homogeneity is advantageous for achieving consistent and predictable results in applications such as drug delivery and catalysis. In addition to size analysis, the zeta potential of K-AuNPs was investigated to assess their colloidal stability (Fig. 3, B) (Qazi et al., 2023). The zeta potential represents the electric charge on the surface of the nanoparticles and is a crucial parameter for understanding their stability and potential interactions with other molecules or surfaces. A zeta potential value of -12 mV was observed for the synthesized K-AuNPs (Fig. 3, B), indicating a strong negative charge on their surface. This negative charge leads to electrostatic repulsion between the nanoparticles, preventing them from aggregating or precipitating in solution (Baker et al., 2020). The stability of K-AuNPs, evidenced by their stable dispersion without clumping or settling, is of great significance for their long-term storage, transport, and biomedical applications (Akhtar et al., 2022).

The comprehensive characterization of K-AuNPs through DLS analysis provides valuable insights into their physical properties and stability. The determined average particle size and narrow size distribution contribute to the understanding of their behaviour and potential applications. Moreover, the negative zeta potential and absence of agglomeration indicate that the K-AuNPs possess excellent colloidal stability. These findings support the potential use of K-AuNPs in various fields, such as nanomedicine, biosensing, and environmental remediation, where stability and controlled behaviour are critical for optimal performance.

### 3.3 Comparative antibacterial activity of K-AuNPs in contrast to pure *Kinnow mandarin* aqueous extract

Infectious diseases caused by microbial pathogens continue to pose a significant challenge to human health, as these organisms have evolved and developed resistance to conventional antibiotics due to their misuse. Recognizing the urgent need for solutions, scientists have turned to alternative systems of medicine for potential breakthroughs. In this study, we conducted a comparative analysis to evaluate the antibacterial properties of crude *Kinnow mandarin* aqueous extract and K-AuNPs (a combination of *Kinnow mandarin* extract and gold nanoparticles) against Gram-negative (*E. coli* and *P. aeruginosa*) and Gram-positive (*B. subtilis*) bacterial strains. The widely recognized disc diffusion method was employed for this analysis.

Remarkably, the crude aqueous extract of *Kinnow mandarin* exhibited impressive antibacterial activity. At a concentration of 300 µg/ml, the extract produced inhibitory zones of 12 ± 0.7, 13 ± 1, and 15 ± 1 against *E. coli, P. aeruginosa,* and *B. subtilis*, respectively (refer to TABLE 1). In comparison, the standard antibiotic Levofloxacin, at the same concentration, showed inhibition zones of 17 ± 0.8, 18 ± 0.7, and 18 ± 0.5 against the respective bacterial strains. However, it was the remarkable performance of the K-AuNPs that truly captured attention, surpassing both the crude *Kinnow mandarin* extract and Levofloxacin. Our findings depicted that K-AuNPs demonstrated an astonishingly high zone of inhibition. Measurements recorded were 21 ± 1.1, 22 ± 0.9, and 23 ± 0.7 against *E. coli, P. aeruginosa,* and *B. subtilis,* respectively. These results highlight the extraordinary potential of harnessing the power of nature in combination with the cutting-edge science of nanoparticles to combat antibiotic resistance. The findings underscore the remarkable antibacterial potency present in *Kinnow* mandarin, a fruit known for its nutritional value and refreshing flavour. Moreover, the integration of gold nanoparticles has introduced a new era of antimicrobial effectiveness, surpassing the limitations of conventional antibiotics. The resounding success of K-AuNPs in inhibiting the growth of both Gram-negative and Gram-positive bacteria brings hope for a future where nature-inspired solutions and nanotechnology converge to address the escalating threat of antibiotic resistance. The significant antibacterial activity exhibited by the *Kinnow mandarin* extract can be attributed to its rich composition of bioactive compounds. Citrus fruits, including *Kinnow* mandarin, are known to contain various phytochemicals such as phenolic acids, flavonoids, and triterpenoids, which possess antimicrobial properties. The aqueous extract derived from the fruit harnesses these compounds, which contribute to its potent antibacterial effects. Additionally, the presence of citric acid and ascorbic acid in citrus fruits enhances the stability and synthesis of nanoparticles, making them ideal candidates for the formation of K-AuNPs. The exceptional antibacterial efficacy of K-AuNPs can be attributed to the synergistic effects of the bioactive compounds present in the *Kinnow mandarin* extract and the unique properties of gold nanoparticles. The combination of these two elements creates a powerful antibacterial agent with enhanced effectiveness. The small size and large surface area of gold nanoparticles provide increased contact with bacterial cells, facilitating efficient antimicrobial action. Furthermore, the presence of bioactive compounds from the *Kinnow mandarin* extract on the surface of the nanoparticles enhances their antimicrobial properties, leading to greater inhibition of bacterial growth.

**Table 1:** Zone of inhibition of *Kinnow mandarin extract*, K-AuNPs and levofloxacin determined against *Escherichia coli, Pseudomonas aeruginosa and Bacillus subtilis.*

Sample (300 µg/ml)	Zone of Inhibition (mm)		
	***Escherichia coli***	***Pseudomonas aeruginosa***	***Bacillus subtilis***
**Control**	**0**	**0**	**0**
**KAPE**	**12 ± 0.7**	**13 ± 1**	**15 ± 1**
**Levofloxacin**	**17 ± 0.8**	**18 ± 0.7**	**18 ± 0.5**
**K-AuNPs**	**21 ± 1.1**	**22 ± 0.9**	**23 ± 0.7**

These findings hold immense significance in the fight against antibiotic resistance. The escalating threat posed by multidrug-resistant bacteria necessitates the exploration of alternative strategies, and the utilization of natural products combined with nanotechnology offers a promising approach. By harnessing the antibacterial potential of *Kinnow mandarin* and augmenting it with the unique properties of gold nanoparticles, we can develop innovative antimicrobial agents that overcome the limitations of conventional antibiotics. This approach not only tackles the issue of antibiotic resistance but also opens avenues for the development of novel therapeutics and infection control strategies. Our comparative analysis demonstrates the exceptional antibacterial activity exhibited by both the crude *Kinnow mandarin* extract and K-AuNPs. While the extract alone shows remarkable potential, the integration of gold nanoparticles significantly enhances its efficacy, surpassing the effectiveness of conventional antibiotics. This study highlights the power of combining nature-inspired solutions with advanced nanotechnology in the battle against antibiotic resistance. The findings emphasize the importance of exploring alternative systems of medicine and integrating them with cutting-edge scientific advancements to address the pressing challenges posed by infectious diseases and antibiotic resistance.

#### 3.3.1 Evaluation of minimum inhibitory concentration of KAPE and K-AuNPs

**Fig. 4:**
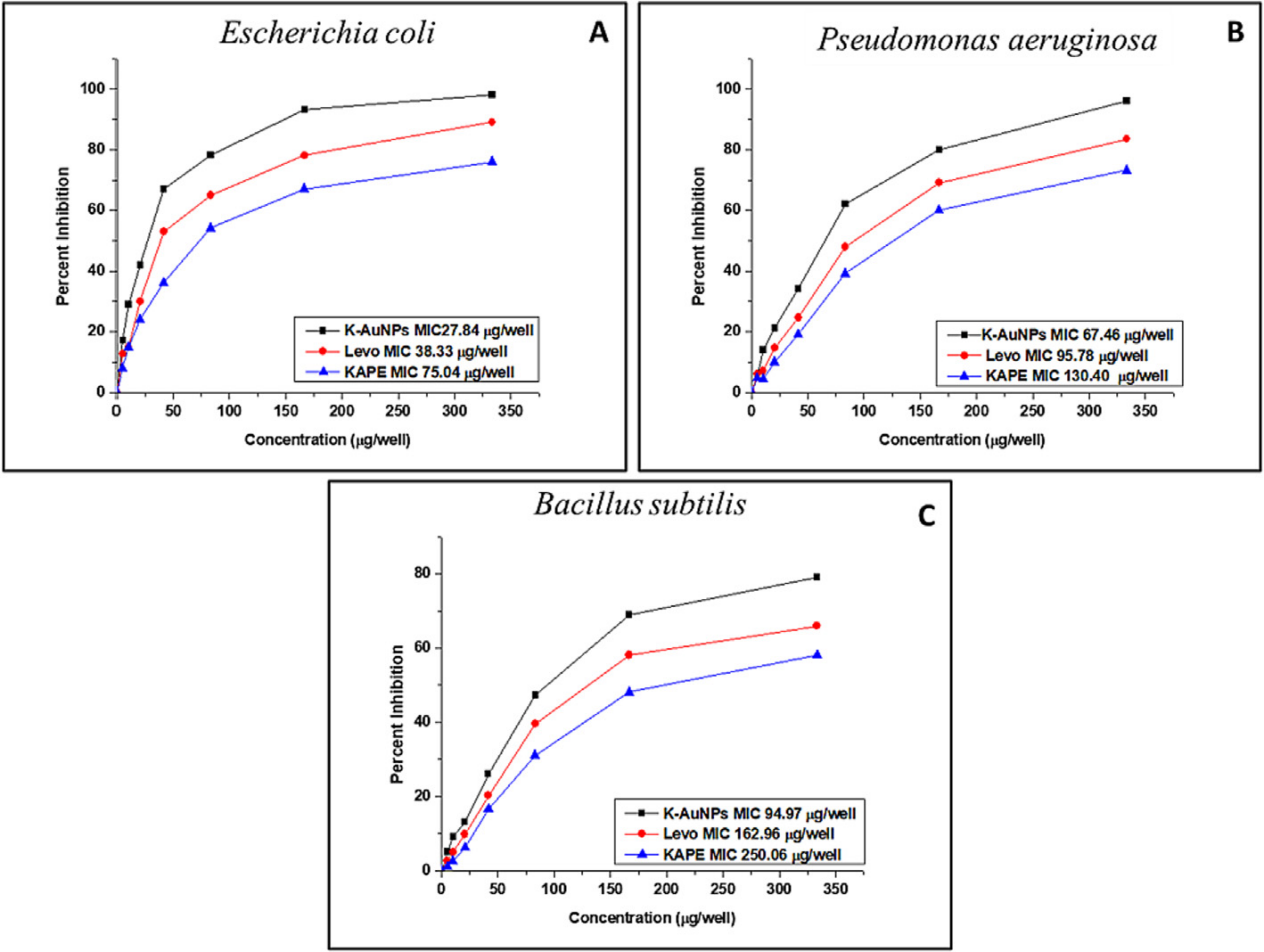
Minimum Inhibitory Concentration of pure KAPE, Levofloxacin and K-AuNPs obtained against *Escherichia coli, Pseudomonas aeruginosa and Bacillus subtilis.* The data represented are average of three determinations obtained under identical experimental conditions.

In our pursuit to combat the escalating threat of antibiotic resistance, we examined the minimum inhibitory concentration (MIC) and MIC50 values of KAPE and K-AuNPs against a range of Gram-negative and Gram-positive bacterial species. The MIC represents the lowest concentration of these agents that completely halts bacterial growth, while the MIC50 indicates the concentration that inhibits 50% of the microbial community. Impressively, our results revealed that *Escherichia coli* exhibited MIC50 values of 27.84 µg/well, 38.33 µg/well, and 75.04 µg/well for K-AuNPs, Levofloxacin, and KAPE, respectively. *Pseudomonas aeruginosa* demonstrated MIC50 values of 67.46 µg/well, 95.78 µg/well, and 130.40 µg/well for the same agents. *Bacillus subtilis* displayed MIC50 values of 94.97 µg/well, 162.96 µg/well, and 250.06 µg/well for K-AuNPs, Levofloxacin, and KAPE, respectively (Fig. 4).

The antibacterial findings of this study and MIC readings portrayed that the encapsulation of *Kinnow mandarin on* AuNPs greatly boosted its effectiveness. K-AuNPs were shown to inhibit the growth of several Gram-negative and Gram-positive bacterial strains at much lower concentrations (Yun and Liu, 2022). The combination of the antibacterial properties of AuNPs and *Kinnow mandarin* extract is believed to exhibit a synergistic effect, explaining the enhanced antibacterial activity observed after encapsulation of the AuNPs with the extract. The antibacterial mechanisms of AuNPs primarily involve two processes: Firstly, they disrupt the membrane potential of bacteria, leading to a decrease in ATPase activity and subsequent reduction in ATP levels. Secondly, they interfere with the binding of components of the ribosome to tRNA. Notably, unlike many bactericidal medications and nanomaterials, AuNPs do not involve reactive oxygen species (ROS)-related pathways in their antibacterial activity (Krishnani et al., 2022).

The exceptional antibacterial activity observed can be attributed to the presence of phytoconstituents in the *Kinnow mandarin* extract. Alkaloids, known for their potent antibacterial action, were found in significant quantities, as supported by recent studies (Kocira et al., 2021). Furthermore, our extracts contained substantial amounts of flavonoids, tannins, saponins, gums, and mucilages, which likely contributed to their remarkable antibacterial properties (Mujeeb et al., 2014).

However, it was the K-AuNPs that emerged as the standout performers, surpassing both the pure *Kinnow mandarin* extract and Levofloxacin at lower dosages, as indicated by our MIC data. The encapsulation of active phytoconstituents in *Kinnow mandarin* with AuNPs resulted in a reduction of *Kinnow mandarin* abundance but a remarkable enhancement in the effectiveness and stability of K-AuNPs. In addition to acting as carriers for *Kinnow mandarin*, the AuNPs themselves exerted antibacterial effects by altering membrane potential, inhibiting ATPase activity, and inducing changes in the ATP level of bacterial cell walls (Mgbeahuruike et al., 2009).

The synergistic antibacterial activity of *Kinnow mandarin* and AuNPs holds great promise, as the combination leads to enhanced efficacy. Furthermore, our extracts contained significant amounts of flavonoids, tannins, saponins, gums, and celluloses, which likely contributed to their exceptional antibacterial properties (Rohela et al., 2016). The multifaceted action of AuNPs, serving both as carriers for *Kinnow mandarin* and as antibacterial agents themselves by modulating membrane potential and ATPase activity, further adds to their potential (Rohela et al., 2016).

Prior research has already demonstrated the antibacterial efficacy of *Kinnow mandarin* extracts, including seeds, against various bacterial species using the agar well diffusion method (Chatterjee et al., 2017). Building upon these findings, our study showcases the immense potential of *Kinnow mandarin* and K-AuNPs as a formidable duo in the battle against bacterial pathogens, heralding a new era where nature-inspired remedies and nanotechnology combine to combat antibiotic resistance.

### 3.4 The anti-diabetic potential of KAPE and K-AuNPs in contrast to standard inhibitor acarbose

#### 3.4.1 α-amylase inhibition potential of KAPE and K-AuNPs

To assess the potential anti-diabetic effect of *Kinnow mandarin* aqueous extract and K-AuNPs, the α-amylase inhibition assay was employed. This assay is commonly used to evaluate the inhibitory activity of compounds against α-amylase, an enzyme involved in carbohydrate metabolism. Acarbose, a known α-amylase inhibitor, was used as a standard inhibitor in this study and exhibited an IC50 value of 45.7 μg/ml. The aqueous extracts of *Kinnow mandarin* and K-AuNPs were tested at various concentrations ranging from 5 to 160 μg/ml. The results showed concentration-dependent inhibition of α-amylase activity by both *Kinnow mandarin* and K-AuNPs.

**Fig. 5:**
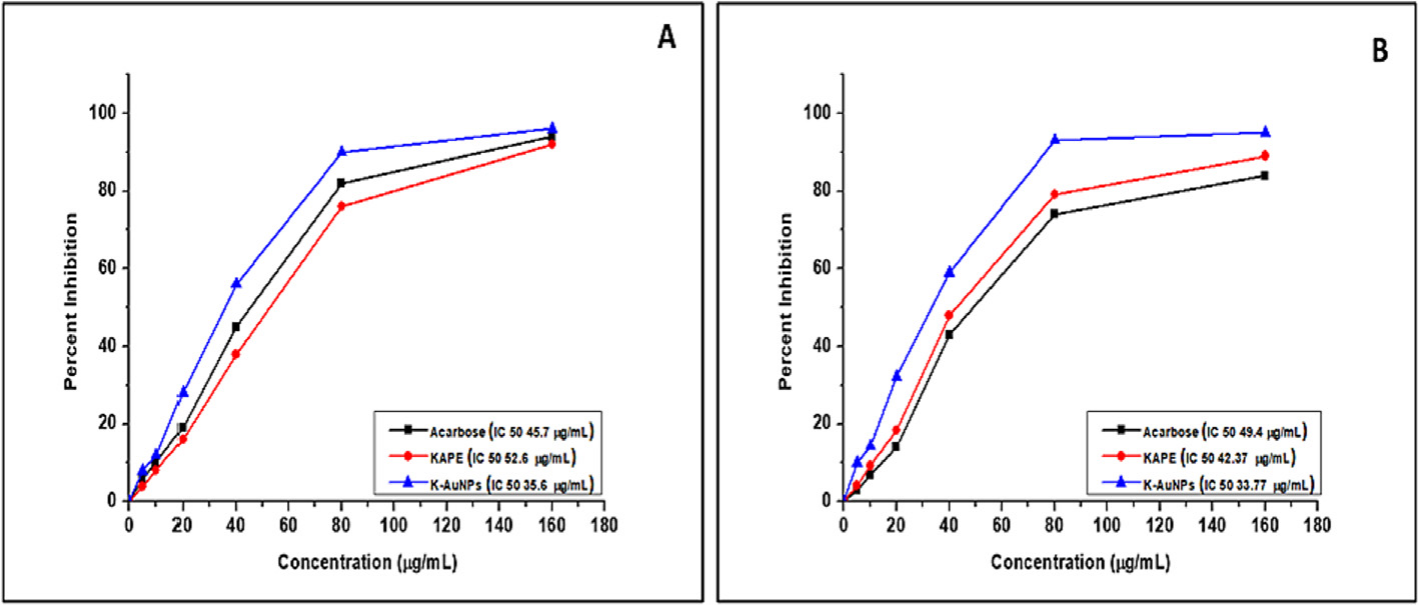
(A)α-amylase inhibition potential of KAPE and K-AuNPs (B) α-glucosidase inhibition potential of KAPE and K-AuNPs. The data represented are the average of three determinations obtained under identical experimental conditions.

For *Kinnow mandarin* extract, the percentage inhibition of α-amylase activity was 4.2%, 8.23%, 16.1%, 38.2%, 76.1%, and 92% at concentrations of 5, 10, 20, 40, 80, and 160 μg/ml, respectively. Similarly, for K-AuNPs, the inhibition percentages were 8.3%, 12.2%, 28.1%, 56%, 90.4%, and 96% at the same concentration range (Fig. 5, A). These results indicate that both *Kinnow mandarin* extract and K-AuNPs possess α-amylase inhibitory activity. Moreover, the percentage inhibition data suggest that K-AuNPs exhibit stronger inhibitory effects compared to the *Kinnow mandarin* extract. The calculated IC50 values for K-AuNPs (35.6 μg/ml) and *Kinnow mandarin* extract (52.6 μg/ml) were lower than that of acarbose (45.7 μg/ml), indicating their potential as effective α-amylase inhibitors (Fig. 5, A).

The significant inhibition of α-amylase activity by K-AuNPs highlights their potential in the development of anti-diabetic agents. These findings support the notion that the encapsulation of *Kinnow mandarin* bioactive compounds onto AuNPs enhances their inhibitory effects, potentially leading to the development of novel therapeutic strategies for managing hyperglycaemia and diabetes mellitus. Further investigations are warranted to elucidate the underlying mechanisms of action and to evaluate the in vivo efficacy and safety profile of K-AuNPs for their potential use in the treatment of diabetes.

#### 3.4.2 α-glucosidase inhibition potential of KAPE and K-AuNPs

To evaluate the inhibitory potential of *Kinnow mandarin* aqueous extract (KAPE) and K-AuNPs against α-glucosidase, an α-glucosidase inhibition assay was performed. This assay is commonly used to assess the ability of compounds to inhibit the activity of α-glucosidase, an enzyme responsible for carbohydrate digestion. The standard inhibitor, acarbose, exhibited an IC50 value of 49.4 μg/ml in this assay. The aqueous extract of *Kinnow mandarin* and K-AuNPs were tested at concentrations of 5, 10, 20, 40, 80, and 160 μg/ml. The results demonstrated concentration-dependent inhibition of α-glucosidase activity by both *Kinnow mandarin* extract and K-AuNPs.

For *Kinnow mandarin* extract, the percentage inhibition of α-glucosidase activity was 4.1%, 9.2%, 18.34%, 48%, 79.2%, and 89% at concentrations of 5, 10, 20, 40, 80, and 160 μg/ml, respectively. Similarly, for K-AuNPs, the inhibition percentages were 10%, 14.3%, 32.2%, 59%, 93.1%, and 95% at the same concentration range (Fig. 5, B). These findings indicate that both *Kinnow mandarin* extract and K-AuNPs possess significant α-glucosidase inhibitory activity. Moreover, the percentage inhibition data suggest that K-AuNPs exhibit stronger inhibitory effects compared to *Kinnow mandarin* extract. The calculated IC50 values for K-AuNPs (33.77 μg/ml) and *Kinnow mandarin* extract (42.37 μg/ml) were lower than that of acarbose (49.4 μg/ml), indicating their potential as effective α-glucosidase inhibitors (Fig. 5, B). These results highlight the antidiabetic potential of *Kinnow mandarin*, as both α-amylase and α-glucosidase are key enzymes involved in carbohydrate metabolism. Moreover, the synergistic effect of coupling *Kinnow mandarin* with K-AuNPs resulted in enhanced inhibitory activity against both α-amylase and α-glucosidase. The successful synthesis of K-AuNPs through the *Kinnow mandarin*-mediated synthesis procedure has further enhanced its antidiabetic properties. The coupling of *Kinnow mandarin* bioactive compounds with K-AuNPs may contribute to improved stability and increased inhibitory effects on α-glucosidase.

These findings suggest that *Kinnow mandarin*, particularly when combined with K-AuNPs, has the potential to be developed into a promising natural anti-diabetic agent with enhanced efficacy and a lower required dosage. Further investigations are warranted to elucidate the underlying mechanisms of action and to evaluate the in vivo efficacy and safety of this combined formulation for potential therapeutic applications in the management of diabetes mellitus.

## 4 Discussion

*Kinnow mandarin* peel extract has garnered significant attention due to its remarkable array of therapeutic properties, including antibacterial, antifungal, anti-inflammatory, antidiabetic, and antiproliferative effects. In this study, we harnessed the potential of *Kinnow mandarin* peel extracts (KAPE) to synthesize gold nanoparticles (K-AuNPs) of substantial size, exhibiting a spherical and monodispersed morphology with exceptional stability.

The crucial findings of our investigation shed light on the intricate process of K-AuNPs synthesis. The active phytoconstituents present in the *Kinnow mandarin* plant extract played a pivotal role in reducing HAuCl_4_ and subsequently fabricating the K-AuNPs. Remarkably, these same phytoconstituents displayed their prowess by effectively stabilizing the nanoparticles, ensuring their longevity and structural integrity. The successful fabrication and synthesis of K-AuNPs were impeccably validated through meticulous TEM analysis, which revealed a particle size of 21 nm (Fig. 2 B).To further validate the phyto-fabrication and spectral properties of K-AuNPs, we conducted UV–visible spectral studies. The resulting spectra showcased a prominent surface plasmon resonance (SPR) band centred at 527 nm, corroborating the presence of K-AuNPs. Intriguingly, two additional bands were observed at 267 and 320 nm, potentially attributable to the characteristic bands of the phytoconstituents within KAPE (Fig. 2, A). The determination of particle size and profile, crucial for understanding the physical characteristics of K-AuNPs, was accomplished through dynamic light scattering (DLS) analysis. The DLS results provided valuable insights, indicating an average particle size of 65 nm (Fig. 3, A), taking into account the hydrodynamic layer surrounding the nanoparticles. Furthermore, the fabrication of K-AuNPs via phytoconstituents demonstrated its stabilizing effect, as evident from the zeta potential analysis, which recorded a highly stable value of -12 mV (Fig. 3, B). These findings underscored the remarkable stability of K-AuNPs, effectively preventing aggregation even after six months of storage at room temperature, a phenomenon consistent with the zeta potential results.

Having characterized KAPE and K-AuNPs, we proceeded to explore their antibacterial and antidiabetic potential. The results unequivocally demonstrated the superiority of K-AuNPs over pure KAPE in terms of antibacterial activity, even when administered at a lower dose. This highlights the heightened antibacterial potential conferred by the unique properties of the synthesized nanoparticles, thereby suggesting their promising role in the treatment of bacterial infections.

Furthermore, the antidiabetic assays revealed the robust α-amylase and α-glucosidase inhibitory activity of KAPE. However, the results also unveiled an even more potent inhibitory potential when KAPE was employed in the fabrication of AuNPs through our innovative synthesis procedure (Fig. 5 A and B). These findings highlight the synergistic effect between KAPE and K-AuNPs, demonstrating an enhanced inhibitory capacity against α-amylase and α-glucosidase enzymes.

The key findings of our study showcase the immense potential of crude *Kinnow mandarin* peel extract and the resulting K-AuNPs in combating bacterial infections and managing diabetes. The phyto-fabrication of K-AuNPs via the unique phytoconstituents of KAPE not only ensures the stability and structural integrity of the nanoparticles but also amplifies their therapeutic effects. These remarkable findings pave the way for the development of novel and effective therapeutic interventions harnessing the power of *Kinnow mandarin* and its synergistic partnership.

## 5 Conclusion

In this study, we achieved a significant milestone by introducing a novel one-pot synthesis technique for producing *Kinnow mandarin* peel phyto-fabricated gold nanoparticles (K-AuNPs). Leveraging the bioactive phyto components within the Kinnow peel, we harnessed their reducing and surface functionalizing properties to successfully synthesize K-AuNPs. These nanoparticles demonstrated remarkable stability due to the abundant attachment of *Kinnow* bioactive phytoconstituents on their surface. A key discovery lies in the elevated antibacterial efficacy observed when pure KAPE was loaded onto AuNPs' surface. Remarkably, even at lower concentrations, the K-AuNPs exhibited superior growth inhibition against both Gram-negative and Gram-positive bacterial strains, outperforming free KAPE and the standard antibiotic levofloxacin. This highlights the potential of K-AuNPs as potent antibacterial agents, allowing for reduced conventional treatment dosages, heightened effectiveness, and minimized toxicity. Our work's significance lies in successfully integrating bioactive components from medicinal plants onto metallic nanoparticles, such as gold. This approach holds promise in minimizing required treatment dosages, leading to more efficient therapeutic interventions and improved patient well-being.

However, to consider K-AuNPs as viable medical drug carriers, further research is crucial. Future investigations should center on evaluating the in vivo activity and safety profile of K-AuNPs to determine their potential for clinical use. These comprehensive studies will offer deeper insights into therapeutic potential and open avenues for innovative treatment strategies. In summary, our study showcases the successful synthesis of K-AuNPs using *Kinnow mandarin* peel extract as a reducing and surface functionalizing agent. The notable stability and enhanced antibacterial efficacy displayed by K-AuNPs underscore their potential as valuable therapeutic agents. This work emphasizes the significance of utilizing phyto-fabricated nanoparticles to optimize treatment outcomes, while also highlighting the necessity for further research to fully unlock K-AuNPs' potential in clinical applications.

## Funding

This research has been funded by the Scientific Research Deanship at the University of Ha’il - Saudi Arabia through project number RG-23 146.

## Institutional Review Board Statement

Not Applicable.

## Declaration of Competing Interest

The authors declare that they have no known competing financial interests or personal relationships that could have appeared to influence the work reported in this paper.
