# Supplementary material for: Exploring Kinnow mandarin's hidden potential: Nature's key to antimicrobial and antidiabetic gold nanoparticles (K-AuNPs)
Source: Saudi J Biol Sci. 2023 Aug 19;30(10):103782. doi: 10.1016/j.sjbs.2023.103782 (PMC10485157; doi:10.1016/j.sjbs.2023.103782)
Supplement: Supplementary data 1 [file mmc1.pdf]

kIN

ORIGINALITY REPORT

14%	11%	9%	1%
SIMILARITY INDEX	INTERNET SOURCES	PUBLICATIONS	STUDENT PAPERS

PRIMARY SOURCES

1	www.mdpi.com<br>Internet Source	3%
2	www.researchgate.net<br>Internet Source	1%
3	mdpi-res.com<br>Internet Source	1%
4	Parvej Ahmad, Sahir Sultan Alvi, Johar Iqbal, M. Salman Khan. "Identification and evaluation of natural organosulfur compounds as potential dual inhibitors of α-amylase and α-glucosidase activity: an in-silico and in-vitro approach", Medicinal Chemistry Research, 2021<br>Publication	1%
5	worldwidescience.org<br>Internet Source	1%
6	link.springer.com<br>Internet Source	1%
7	oaji.net<br>Internet Source	<1%
